# Convolutional Neural Network-Based Classification of Driver’s Emotion during Aggressive and Smooth Driving Using Multi-Modal Camera Sensors

**DOI:** 10.3390/s18040957

**Published:** 2018-03-23

**Authors:** Kwan Woo Lee, Hyo Sik Yoon, Jong Min Song, Kang Ryoung Park

**Affiliations:** Division of Electronics and Electrical Engineering, Dongguk University, 30 Pildong-ro 1-gil, Jung-gu, Seoul 100-715, Korea; leekwanwoo@dgu.edu (K.W.L.); yoonhs@dongguk.edu (H.S.Y.); whdwhd93@dongguk.edu (J.M.S.)

**Keywords:** aggressive driving emotion, near-infrared light camera sensor, thermal camera sensor, convolutional neural network

## Abstract

Because aggressive driving often causes large-scale loss of life and property, techniques for advance detection of adverse driver emotional states have become important for the prevention of aggressive driving behaviors. Previous studies have primarily focused on systems for detecting aggressive driver emotion via smart-phone accelerometers and gyro-sensors, or they focused on methods of detecting physiological signals using electroencephalography (EEG) or electrocardiogram (ECG) sensors. Because EEG and ECG sensors cause discomfort to drivers and can be detached from the driver’s body, it becomes difficult to focus on bio-signals to determine their emotional state. Gyro-sensors and accelerometers depend on the performance of GPS receivers and cannot be used in areas where GPS signals are blocked. Moreover, if driving on a mountain road with many quick turns, a driver’s emotional state can easily be misrecognized as that of an aggressive driver. To resolve these problems, we propose a convolutional neural network (CNN)-based method of detecting emotion to identify aggressive driving using input images of the driver’s face, obtained using near-infrared (NIR) light and thermal camera sensors. In this research, we conducted an experiment using our own database, which provides a high classification accuracy for detecting driver emotion leading to either aggressive or smooth (i.e., relaxed) driving. Our proposed method demonstrates better performance than existing methods.

## 1. Introduction

Aggressive driving causes most car accidents and accounts for the largest percentage of fatal crashes [1]. For such a serious problem, detection of aggressive driving has been relegated mainly to traffic police officers. Because there are too few police officers to monitor every road simultaneously [2], intelligent surveillance systems are clearly needed. Most previous studies have attempted to detect the drivers’ aggressive behaviors by observing vehicle movement using accelerometers and gyro-sensors installed on a smart phone [2,3,4,5,6]. Although not aimed at detecting aggressive driving, there was also a study on driving behavior using steering wheel angles as the input and output [7]. In addition, some works focused on fatigue or drowsy driver detection. Others used the drivers’ facial features [8,9,10,11], voice signals, car–voice interactions [12,13], or bio-signals [11,14,15,16] to help recognize up to six types of emotions.

Extant aggressive-driver detection methodologies are deficient for two reasons. First, the construction of algorithms to extract complex facial feature points and accurate classification into either smooth (i.e., relaxed) or aggressive driving has proven difficult. Secondly, we lack adequate hardware to accurately and quickly classify large amounts of visual data. However, recent algorithmic and hardware developments indicate that intelligent surveillance systems are near-at-hand, and can be used for human emotion recognition in complex environments. Notably, the application of convolutional neural networks (CNN) has significantly improved the accuracy of human emotion recognition [17,18,19]. Recently, more advanced methods based on the 2D or 3D inception-ResNet model [20,21] and you only look once (YOLO) [22] were also used for facial expression recognition. Therefore, we decided to research CNN-based aggressive driving detection using near-infrared (NIR) light and thermal cameras. In Section 2, previous studies are reviewed to reveal opportunities and limitations.

## 2. Related Works

Previous studies on vehicle movement mostly utilized accelerometers and gyro-sensors built into smart phones for detecting driving behaviors [2,3,4,5,6]. The experimental methods that used gyro-sensors and accelerometers involved the following: First, a two-axis accelerometer, built into a smart phone, was used, and patterns of acceleration were classified into six abnormal driving behavior types. Then, a support vector machine was used to detect abnormal driving [3]. Another study utilized accelerometers and gyro-sensors to divide abnormal driving patterns into unsafe left and right turns, lane departures, sudden braking, and speeding-up. A dynamic time-warping algorithm, which compares two temporarily consecutive data sets and judges their similarity, was implemented to evaluate the difference in patterns. A Bayesian classifier was then applied to classify the driving behaviors [4]. Another method used a two-axis accelerometer and applied fuzzy logic to obtain *x*- and *y*-axis scores of four driving behaviors (i.e., take off, brake, turn left, and turn right) measured on a bus. Through this method, reckless driving could be detected [5]. The Gaussian mixture model (GMM) classifier was also used to categorize driving behaviors of both young and senior drivers. The GMM classifier used gyro-sensor values as inputs [6]. These methods used accelerometers and gyro-sensors built into smartphones and did not demand any separate equipment. The sensors were portable and collected real-time data. Having been integrated into a smart phone, researchers eliminated the need for complicated installation of networked sensors. Despite these advantages, if the vehicle operated in a GPS-deprived area, or if the GPS receiver demonstrated poor performance, accurate values could not be utilized. Thus, aggressive driving remained difficult to detect. Additionally, driving on a mountain road and other winding-type roads could be misrecognized as aggressive driving, owing to the quick and irregular turns and frequent braking.

Other studies used new techniques to recognize driver emotions [8,12,13,14,16] and to monitor fatigue [9,10,11,15]. These researchers recognized or judged a driver’s emotional or physiological state using microphones, recording the driver’s status as audio signals, or electrocardiograms (ECG) and electroencephalography (EEG), storing bio signals. Video recording equipment (e.g., NIR light or thermal cameras) have also been used. There have also been studies that examined a driver’s voice or car–voice interactions [12,13]. Another group measured bio-signals (e.g., ECG, EEG, pulse) to correlate emotions or sleepiness to abnormal driving behaviors [14,15,16]. Whereas bio-signals are useful to directly identify a driver’s emotional or physiological responses, the required equipment is expensive and must be attached directly to the driver’s skin. Accordingly, the driver may experience discomfort. Additionally, any violent motions could detach sensors, making it very difficult to get accurate measures. These problems were overcome by using camera-based emotion studies. Visible light cameras, NIR cameras, and thermal cameras have been used to obtain drivers’ facial images, which are then analyzed as input signals. From such analyses, driver fatigue and emotional state can be identified, potentially detecting abnormal driving [8,9,10]. There was a study that used only a thermal camera to recognize a driver’s emotion [8]. Color-based detection, morphology-based detection, and region growing-based detection were applied to the images obtained by the thermal camera. An AND operation was applied to the outcomes of the three methods to determine the face region, from which features were extracted through a histogram of oriented gradients (HOG). Later, an experiment was conducted to classify six basic emotional states using the modified Hausdorff distance. However, the average classification accuracy was only about 66.8%. Alternatively, two NIR cameras and one visible light camera were used for fatigue-driving detection, based on human physical conditions [9,10]. The percentage of eye-closure over time and the average eye-closure speed were measured to judge driver fatigue [9]. Driver fatigue was also measured by designating the mouth as the region of interest (ROI) to detect yawning [10]. Regarding the NIR camera, the difference between abnormal and normal driving situations can be intuitively identified from its input images. Alternatively, a thermal camera can also detect subtle changes in temperature, which could be attributed to a driver’s emotional state. Recently, camera images and audio signals have been applied for the detection of driver fatigue and emotion. Such an application has not yet been extended to distinguish between a driver’s aggressive and smooth driving states. Research on driver emotion [8] has not considered its relationship with aggressive driving. Moreover, because complex emotions can be involved in aggressive driving, there has been no reliable method to match trigger emotions to aggressive driving. Camera-based methods have used a single camera type. Consequently, it could not detect aggressive driving under different situations. Table 1 shows the summarized comparisons of our method and previous works.

## 3. Motivation and Contributions

In view of the problems and constraints of previous works explained in Section 2, our research proposes a CNN-based method for detecting a driver’s aggressive driving emotion using facial images obtained from both NIR and thermal cameras. Our research is novel in the following four ways.

- This research is the first CNN-based attempt to classify aggressive and smooth driving emotions using nonintrusive multimodal cameras (i.e., NIR and thermal cameras) and facial features as inputs.
- From NIR images, both the eyes and mouth, which show the most remarkable changes of facial expression, are extracted and converted into 3-channel images and used as inputs to the first CNN. From thermal images, the middle-forehead and cheek areas, which show the largest temperature changes, are extracted and converted into 3-channel images, to be used as inputs to the second CNN. The two CNN output scores are then fused at the score-level to enhance the classification accuracy for aggressive and smooth driving emotions.
- From 15 subjects carrying out aggressive and smooth driving experiments, a total of 58,926 images were obtained using NIR and thermal cameras. The images were used to intensively train the CNN. Thus, CNN-based classification of aggressive and smooth driving emotion becomes more accurate and robust over time, owing to correlating changes in drivers’ emotional status.
- A database of driver images is constructed in this research, using NIR and thermal cameras and a trained CNN model. It is open to other researchers to ensure impartial performance evaluation.

The remainder of this paper is organized as follows: Section 4 explains the multimodal cameras and CNN-based method for detecting aggressive driving. Section 5 and Section 6 present the experimental results and conclusions, respectively.

## 4. Proposed Method for CNN-Based Detection of Aggressive Driving Emotion

### 4.1. Proposed Device, Experimental Setup, and Method

**Figure 1:**
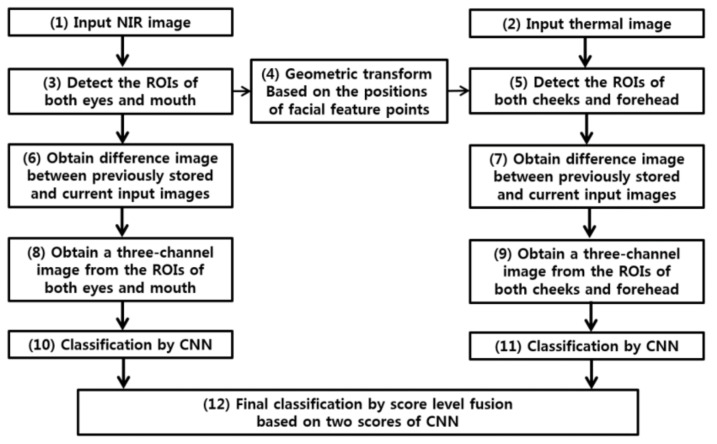
Flowchart of proposed method.

Figure 1 shows the proposed emotion classification for smooth and aggressive driving.

**Figure 2:**
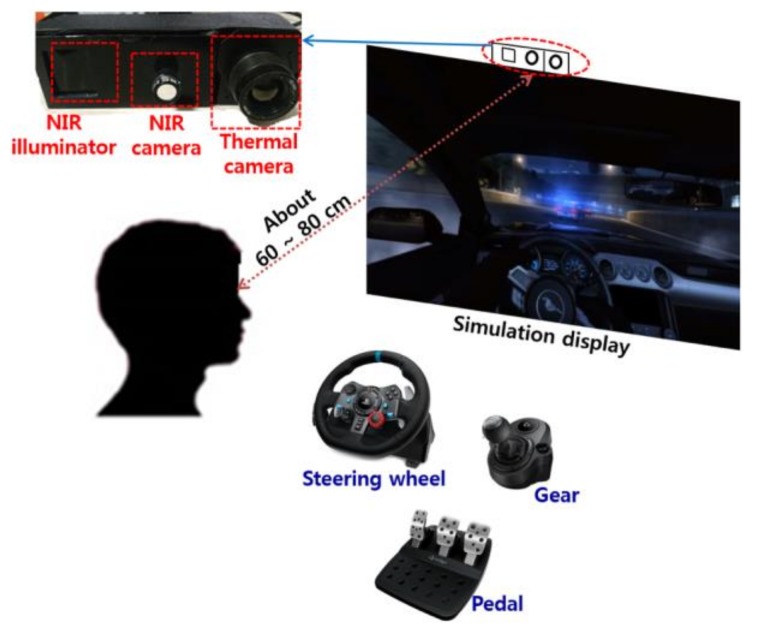
Experimental setup for classifying aggressive and smooth driving emotion using multimodal cameras.

**Figure 3:**
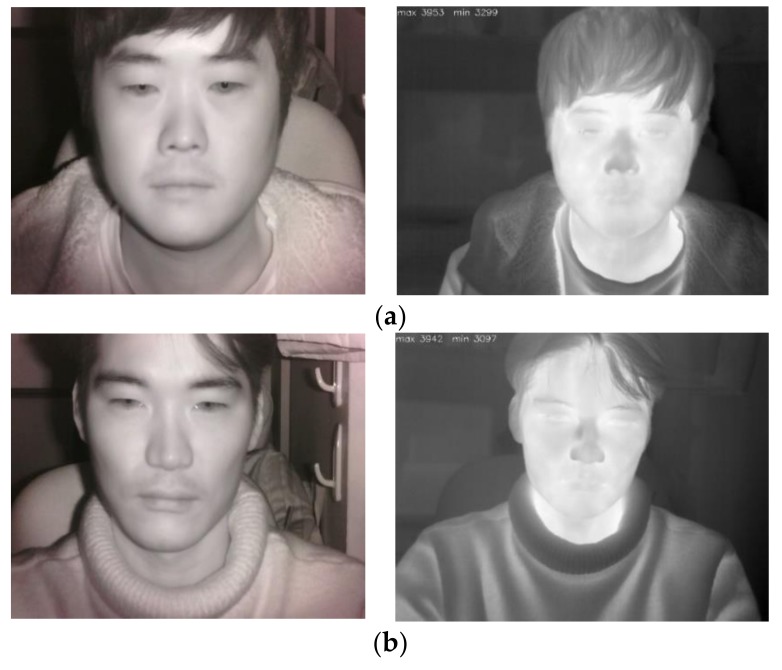
Examples of captured images by NIR (left images) and thermal (right images) cameras from (**a**) person 1 and (**b**) person 2.

**Figure 4:**
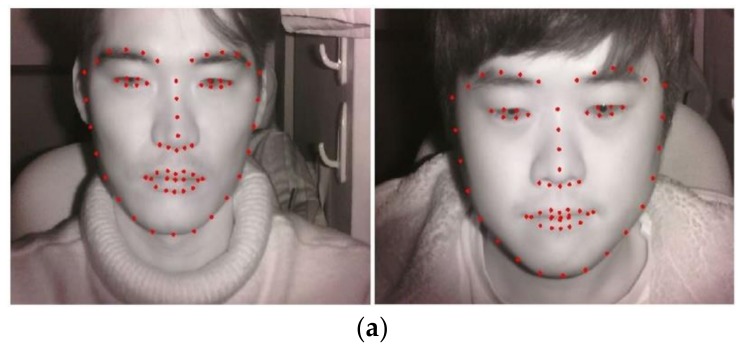

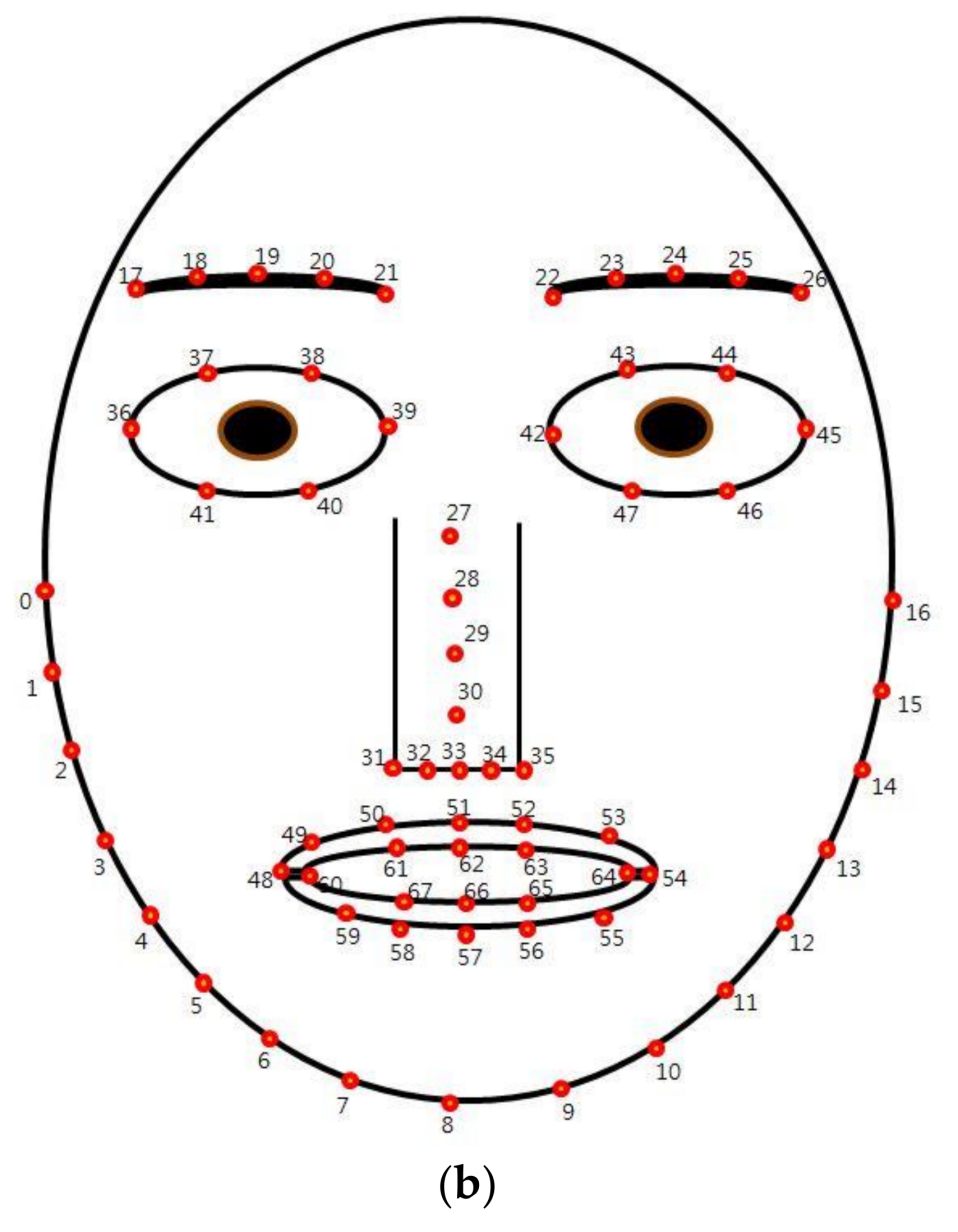
Examples of (**a**) detected facial feature points; and (**b**) the index numbers of facial feature points.

As shown in steps (1) and (2) of Figure 1, the facial images of a user are simultaneously obtained by NIR and thermal cameras. Figure 2 illustrates the experimental setup. NIR and thermal multimodal cameras were installed atop a 24-inch monitor, which displays a driving simulator, and are used to obtain facial images of drivers (see Figure 3). Because experimenting in a real car is dangerous, we utilized two types of driving simulators to create aggressive and smooth driving conditions. As shown in Figure 2, every subject used a steering wheel, gear shifter, and pedals to simulate real driving. Simulator details are discussed in Section 5.1. NIR and thermal cameras were used for data acquisition. Thermal energy from a person can be measured by long-wave IR (LWIR) and medium-wave IR (MWIR) lights [23]. Thus, the images taken in these sub-bands are called “thermal images.” This research used a Tau2 FLIR thermal camera, which has a spectral range of 7.5–13.5 μm, like most LWIRs, including the upper MWIR range. The scene range (i.e., high gain) was from −25 °C to +135 °C [24]. Additionally, the NIR camera used in the experiment was manufactured by ELP-USB500W02M-L36 [25]. An 850 nm NIR band-pass filter was attached to receive light in the NIR range [26]. An NIR illuminator was fabricated, consisting of six NIR light emitting diodes (LED), each with an 850-nm wavelength [27]. Thus, each NIR image obtained 640 × 480 pixels of 8 bits each, and the thermal image had 640 × 512 pixels of 14 bits each. In Step (3) of Figure 1, d-lib facial feature tracker was used for NIR images [28] to extract 68 feature points, as shown in Figure 4. Based on the extracted feature points, ROI was designated to classify the emotional states of aggressive and smooth driving.

**Figure 5:**
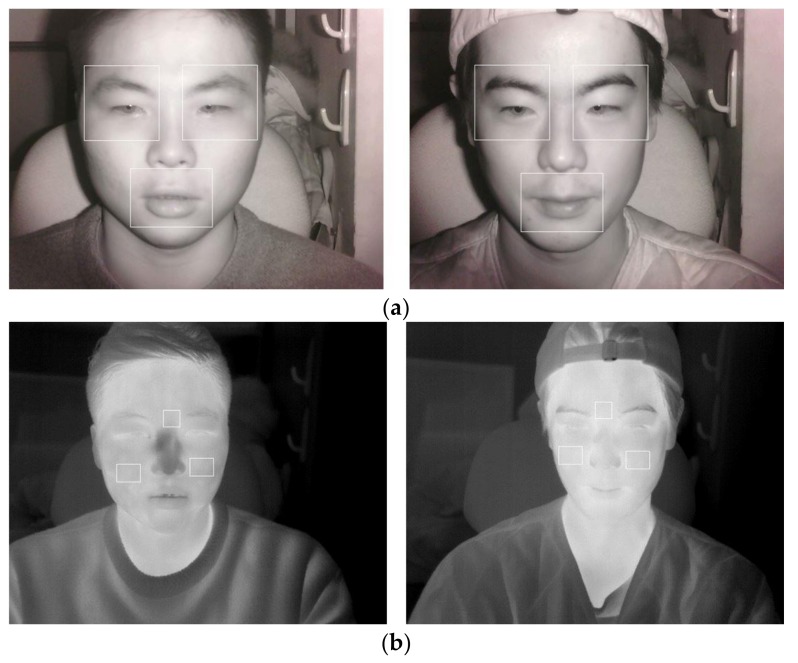
ROI regions in (**a**) NIR image; and (**b**) thermal image.

According to Ekman’s facial action coding system [29], emotional changes appear mainly in the mouth and eye regions, as shown in Figure 5a. The ROI for the left eye was formed around facial feature point 39. For the right eye, it was formed around point 33 (see Figure 4b). The multimodal camera developed in this research is 10.5 × 5.5 × 3.7 cm^3^ in width, height, and depth, respectively, as shown in Figure 2. Having such a small size, it can be installed on the dashboard or near the front sun shade. The distance from this device to the driver’s face differs per sitting height. This fact was mitigated by modifying the distance from 60 cm to 80 cm in the current experiment, as shown in Figure 2. Therefore, the face size in the input NIR and thermal images changed, as seen in Figure 4a. To prevent ROI from being affected by the size change, we adaptively measured ROI width and height based on the distance between facial feature points, 0 and 16, as shown in Figure 4b. Thus, each ROI adapted robustly to the changes in a user’s face. As shown in Step (4) of Figure 1, the NIR image feature points positions were translated to those of the thermal image via geometric transform, as shown by Equations (1) and (2) [30].

(1) $$\left[\begin{array}{cccc} T_{x0} & T_{x1} & T_{x2} & T_{x3}\\ T_{y0} & T_{y1} & T_{y2} & T_{y3}\\ 0 & 0 & 0 & 0\\ 0 & 0 & 0 & 0 \end{array}\right]=\left[\begin{array}{cccc} a & b & c & d\\ e & f & g & h\\ 0 & 0 & 0 & 0\\ 0 & 0 & 0 & 0 \end{array}\right]\left[\begin{array}{cccc} V_{x0} & V_{x1} & V_{x2} & V_{x3}\\ V_{y0} & V_{y1} & V_{y2} & V_{y3}\\ V_{x0}V_{y0} & V_{x1}V_{y1} & V_{x2}V_{y2} & V_{x3}V_{y3}\\ 1 & 1 & 1 & 1 \end{array}\right]$$

(2) $$\left[\begin{array}{c} \begin{array}{c} {T^{\prime }}_{x}\\ {T^{\prime }}_{y} \end{array}\\ \begin{array}{c} 0\\ 0 \end{array} \end{array}\right]=\left[\begin{array}{c} \begin{array}{cc} a & b\\ e & f \end{array}\;\;\;\;\begin{array}{cc} c & d\\ g & h \end{array}\\ \begin{array}{cc} 0 & 0\\ 0 & 0 \end{array}\;\;\;\;\begin{array}{cc} 0 & 0\\ 0 & 0 \end{array} \end{array}\right]\left[\begin{array}{c} \begin{array}{c} {V^{\prime }}_{x}\\ {V^{\prime }}_{y} \end{array}\\ \begin{array}{c} {V^{\prime }}_{x}{V^{\prime }}_{y}\\ 1 \end{array} \end{array}\right]$$

where (*V_x_*_0_, *V_y_*_0_), …, (*V_x_*_3_, *V_y_*_3_) show the four positions in the visible light image. (*T_x_*_0_, *T_y_*_0_), …, (*T_x_*_3_, *T_y_*_3_) show the corresponding four points in the thermal image. Based on the obtained eight parameters of *a*, *b*, *c*, …, *h* from Equation (1), the positions, (*T*′*_x_*, *T*′*_y_*) can be computed by Equation (2). To obtain these eight parameters, the calibration method based on [30] was performed. After the positions of facial feature points were obtained from the thermal images, as in Step (5) of Figure 1, both cheeks and the middle of the forehead were designated as ROIs (see [30]). Figure 5b presents examples of the designated ROIs. The ROI of the left cheek is specified around the center between facial feature points 31 and 2 in Figure 4b, whereas the right cheek ROI is formed at the center between points 35 and 14 in Figure 4b. The ROI for the middle of the forehead is designated to be at the center between points 21 and 22 in Figure 4b. The width and height of each ROI were adaptively determined based on the distance between facial feature points 0 and 16, shown in Figure 4b, to enhance the robustness of ROI to the changing distance from the camera device to the driver’s face.

Next, images were extracted from three ROIs of the NIR image. The difference images were obtained from the extracted images, plus those extracted from the three ROIs of the initial NIR image of the driver (Step (6) in Figure 1). Although this cannot be valid in a real-world scenario, we assumed, in our experimental setup, that the initial NIR image of the driver could be acquired when he started the car, because, at this moment, a smooth driving emotion will be shown. Therefore, when a difference image for the initial NIR image is obtained, the variations indicating aggressive driving emotion can be deduced. Next, images were extracted from the three ROIs of the thermal image. The difference images were obtained from the extracted images plus those extracted from three ROIs of the initial thermal image of the driver (Step (7) in Figure 1). Each difference image was resized to 224 × 224 pixels to be used as a CNN input. The reason for this resizing process will be explained in Section 4.2. Three difference images were then obtained from the NIR image to form a 3-channel image (Step (8) of Figure 1). Another was obtained from the thermal image (Step (9) in Figure 1). Two 3-channel images were used as CNN inputs (Steps (10) and (11)), and the scores of two CNNs were fused at the score-level, thereby judging aggressive and smooth driving emotions (Step (12) in Figure 1). The CNN structure used in this research is explained in the following section.

### 4.2. CNN Structure

Table 2 shows the CNN structure adopted by this research. We used the VGG face-16 model. The VGG face-16 model uses the same structure as the VGG Net-16 model [31], but the VGG face-16 model was originally trained with huge numbers of face images (not by images for object recognition) [32]. In addition, the pre-trained VGG face-16 model was finely tuned by our training data (fine-tuning), and this was used for our experiment. Therefore, we can assume that the VGG face-16 model used in our experiment shows the features for face representation.

Because the pre-trained VGG face-16 model is fine-tuned, the input data images were resized to 224 × 224 pixels via bilinear interpolation. The VGG face-16 model consists of 13 convolutional layers, five pooling layers, and three fully connected layers (FCL).

The feature map size obtained from the first convolutional layer was 224 × 224 × 64. The height and width were calculated based on [33]. The output feature map (OF*_k,l,n_*) for a standard convolution, based on stride 1 and padding, is generally obtained by the input feature map (IF*_k+i_*_−1*,l+j*−1*,m*_) and convolution kernel (K*_i,j,m,n_*) as [34]

OF*_k,l,n_* = Σ*_i,j,m_* (K*_i,j,m,n_* · IF*_k+i_*_−1*,l+j*−1*,m*_) (3)

Based on Equation (3), the computational cost is dependent on the multiplicatively of the number of output channels, the input feature map size, the kernel size, and the number of input channels, and [34]. As seen in Equation (4) (*x* and *y* are the input and output), every convolution layer was connected to a rectified linear unit (ReLU) layer. The ReLU can also remove the back propagation vanishing gradient problem during training, and it can reduce the training time [26,35,36,37].

(4) y = max(0,x)

After the second, fourth, seventh, 10th, and 13th convolutional layers with the ReLU, a max pooling layer followed. The max pooling layer uses the maximum value in the filter with a specified size and conducts subsampling. After 13 convolutional layers, 13 ReLU layers, and five max pooling layers, the ultimate feature map size was 7 × 7 × 512 pixels. Additionally, data passed through three FCLs. The output nodes of the first, second, and third FCLs were 4096, 4096, and 2, respectively. Because this research aimed to distinguish two classes driving emotions (smooth and aggressive), the third FCL was composed of two nodes. Generally, a CNN becomes too dependent on training data (i.e., “over-fitting”). To solve this problem, we used dropout methods based on a dropout probability of 50% [31,35,38]. In the third FCL, a softmax function was applied [39].

### 4.3. Score-Level Fusion of the Outputs of Two CNNs

As shown in Step (12) of Figure 1, this research conducted score-level fusion for the CNN output scores of NIR images (i.e., *S*_1_ of Equations (5) and (6)) and thermal images (i.e., *S*_2_ of Equations (5) and (6)). The final score was used to classify smooth and aggressive driving emotions. Weighted SUM and PRODUCT rules were compared, as seen in Equations (5) and (6).

(5) $$WS=w_{1}S_{1}+\;w_{2}S_{2}$$

(6) $$WP=d_{1}{}^{S_{1}}d_{2}{}^{S_{2}}$$

*WS* and *WP*, respectively, are the scores by weighted SUM and weighted PRODUCT rules. $S_{i}$ is the CNN output score, and $w_{i}$ is the weight. Among weighted SUM and weighted PRODUCT rules, the optimal rule with optimal weights was determined to have the least error in classifying smooth and aggressive driving emotions via only training data.

## 5. Experimental Results

### 5.1. Experimental Scenario, Environment and Data

We used 15 subjects (10 Koreans, 2 Vietnamese, 2 Pakistanis, and 1 Mongolian) between ages of 25 to 34 years, in the experiment. Eight subjects were male and the remaining seven were female. All the subjects voluntarily participated in our experiments. Before the experiments, all the participants were provided with sufficient explanations, including the purpose and procedure of our experiments, and how the experimental data would be used, etc. Then, we obtained written consents from all of the participants before experiments.

**Figure 6:**
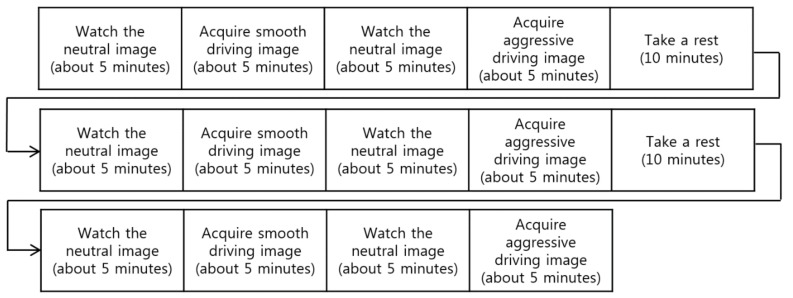
Experimental procedure. Smooth and aggressive driving images refer to images acquired while the participant is operating the smooth and aggressive driving simulators, respectively.

Because it was too risky to create an aggressive driving situation under real traffic conditions, we utilized two types of driving simulator, as shown in Figure 2, to assess baseline aggressive and smooth driving situations. As illustrated in Figure 6, the experiment included 5 min of smooth driving and another 5 min of aggressive driving. Between each section of the experiment, every subject watched a sequence of neutral images from the international affective picture system [40], thereby maintaining neutral emotional input. After the experiment, the subjects rested for about 10 min. This procedure was repeated three times. Figure 6 shows one example from the experimental procedure. The order of “acquiring smooth driving image (about 5 min)” and “acquiring aggressive driving image (about 5 min)” were randomly changed for different participants (without notification in advance) in order to prevent the experimental results from being biased by the order. The temperature and illumination were set to about 26 °C and 385 lux, respectively.

The autonomous driving mode of Euro Truck Simulator 2 [41] was selected as the smooth driving simulator, because it was the most appropriate. For the simulation game involving the acquisition of input from aggressive driving, the competition mode of Need for Speed (Deluxe Edition) [42] was used, because it was the most suitable. We aimed to measuring real emotion (not disguised or not pretended expression) of the drivers. Therefore, we did not collect our data from actors. Our goal was to measure the actual emotions of drivers in environments most similar to actual driving situations. Therefore, to induce aggressiveness in the subjects by causing them to make mistakes or introducing another person into the simulation which would induce aggressive behavior is different from actual driving situations. Because it was too risky to create an aggressive driving situation under real traffic conditions, we utilized two types of driving simulator. The two simulators of the autonomous driving mode of Euro Truck Simulator 2 and the competition mode of Need for Speed (Deluxe Edition) have been widely used for experiments because they can represent the actual driving environment. Therefore, we performed the experiments on these two simulators.

In this experiment, a Samsung S24C450 24-in monitor [43] was used. As shown in Figure 2, the distance from a user to the monitor and the dual camera device was 60–70 cm.

Table 3 presents the database used in this research. Two-fold cross validation was conducted for the performance evaluation as follows: During the first validation, a sub-data set of eight subjects was used for training, and that of the remaining seven was used for testing. Alternatively, during the second validation, a sub-data set of seven subjects was used for training, and that of the remaining eight was used for testing.

For CNN training and testing, we used a desktop computer with an Intel^®^ Core™ i7-3770 CPU @3.50 GHz (Intel Corp., Santa Clara, CA, USA) [44], 16-GB memory and an NVIDIA GeForce GTX 1070 (Intel Corp., Santa Clara, CA, USA) (i.e., computer-unified device architecture cores of 1920) with memory of 8 GB [45]. The CNN algorithm was implemented using Windows Caffe (Version 1) [46]. We have opened our thermal and NIR camera database (i.e., Dongguk aggressive and smooth driving database (DASD-DB1)) and the trained CNN model to other researchers (see [47]) for fair performance evaluation.

### 5.2. Features of NIR and Thermal Images and the Comparison of Performance

As mentioned in Section 4.1, images were extracted from three ROIs of a NIR image (see Figure 5a). The difference images were acquired using these extracted images and others were extracted from three NIR image ROIs, taken while a driver watched the initial neutral image shown in Figure 6. Additionally, images were extracted from three ROIs of the thermal image shown in Figure 5a, and the difference images were acquired from these extracted images and the images extracted from three ROIs of the initial thermal image, taken when a driver watched the initial neutral image of Figure 6. We compared the ROI features with other drivers’ facial image features to verify the effectiveness of classifying smooth and aggressive driving emotions. For a comparison of performance, we conducted a *t*-test [48] and Cohen’s *d* analysis [49,50] against the difference features of the initial images obtained while a driver watched the neutral image shown in Figure 6 and those of images taken during smooth and aggressive driving situations. The null hypothesis for the *t*-test assumed that there was no difference among features of images in smooth and aggressive driving situations [48]. If the *p*-value was 0.05 or below in the *t*-test, the null-hypothesis was rejected at a 95% confidence level, indicating a difference in features between smooth and aggressive driving at a 95% confidence level. If the *p*-value was 0.01 or below, the null-hypothesis was rejected at a 99% confidence level, indicating a difference in features between smooth and aggressive driving at a 99% confidence level. Therefore, as the *p*-value decreases, the difference between two measured datasets increases at a statistically significant level.

**Figure 7:**
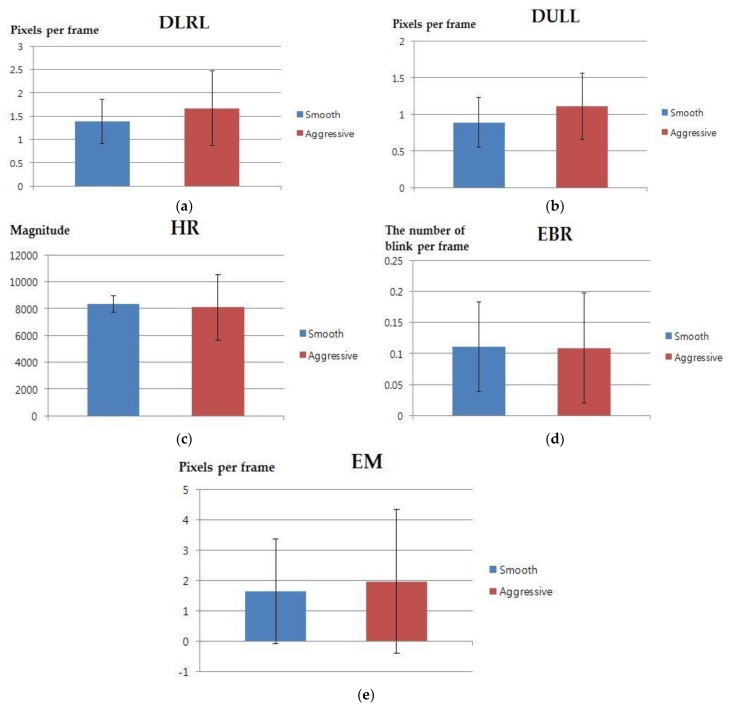
Graphs of mean and standard deviation for 5 features between smooth and aggressive driving (**a**) euclidean distance change between left and right lip corners (DLRL); (**b**) euclidean distance change between upper and lower lips (DULL); (**c**) facial temperature-based heart rate (HR); (**d**) eye-blinking rate (EBR); (**e**) eyebrow movement (EM).

**Figure 8:**
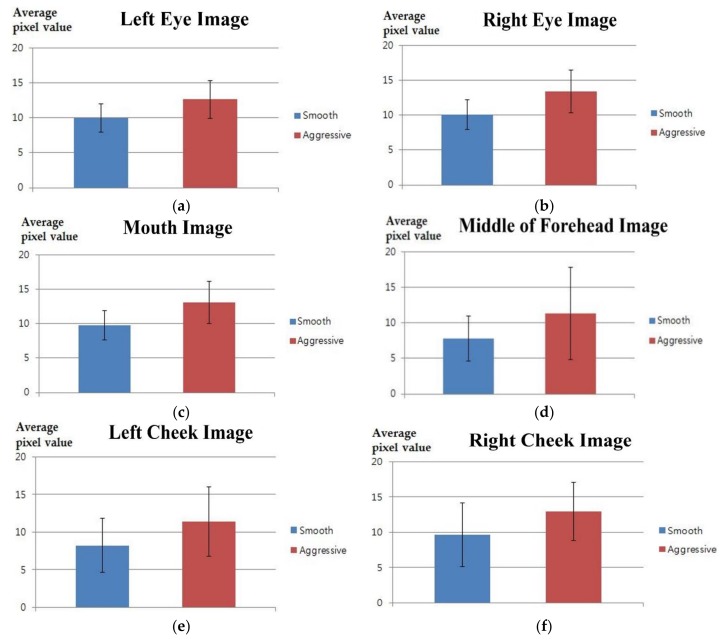
Graphs of means and standard deviations for pixel values of NIR and thermal ROIs between smooth and aggressive driving. ROIs of (**a**) left eye; (**b**) right eye; (**c**) mouth; (**d**) middle of forehead; (**e**) left check; and (**f**) right cheek.

Other features, extracted from facial images, were used introduced in [51]. Table 4 and Figure 7 present the mean, standard deviation, *p*-value, Cohen’s *d* value, and effect size for the five feature values. Table 5 and Figure 8 show the means, standard deviations, *p*-values, Cohen’s *d* values, and effect sizes for the feature values used in Figure 5. In Table 4 and Table 5 and Figure 7 and Figure 8, “smooth” and “aggressive” indicate smooth driving and aggressive driving, respectively. In Table 5 and Figure 8, the left eye, right eye, and mouth come from three ROIs of the NIR image shown in Figure 5a. Whereas, the middle of forehead, left cheek, and right cheek are from three ROIs of the thermal image shown in Figure 5b.

As shown in Table 4 and Table 5 and Figure 7 and Figure 8, the *p*-value for the pixel values of NIR and thermal ROIs between smooth and aggressive driving was lower than that of the other five features. As shown in Table 5, the *p*-values of the left eye and mouth ROIs of the NIR image were 0.0046 and 0.0021, respectively, which are lower than 0.01. These features were reliable for classifying smooth and aggressive driving at a 99% confidence level. The remaining four features were reliable at 95% confidence levels. Additionally, as shown in Table 4 and Table 5, Cohen’s *d* value, between smooth and aggressive driving of the pixel values in NIR and thermal ROIs, wais larger than those of the five features. In the case of the pixel values of NIR and thermal ROIs, as shown in Table 5, the effect size was large in every case. Consequently, six *p*-values of NIR and thermal ROIs turned out to be more effective for identifying the difference between smooth and aggressive driving.

### 5.3. Training of CNN Model

We used the stochastic gradient descent (SGD) [52] method for training. SGD defines the division of the training set via mini-batch size iterations and specifies the operation of training duration as 1 epoch. Training occurs for a predetermined number of epochs. We used the following parameters for the SGD method: base learning rate = 0.0001, gamma = 0.1 (i.e., drop learning rate factor), batch size (i.e., mini-batch size) = 20, momentum = 0.9, weight decay = 0.0005, and epoch = 30. The weights used were initially set based on Gaussian random distribution (mean = 0 and standard deviation of 0.01 with the biases of 0).

**Figure 9:**
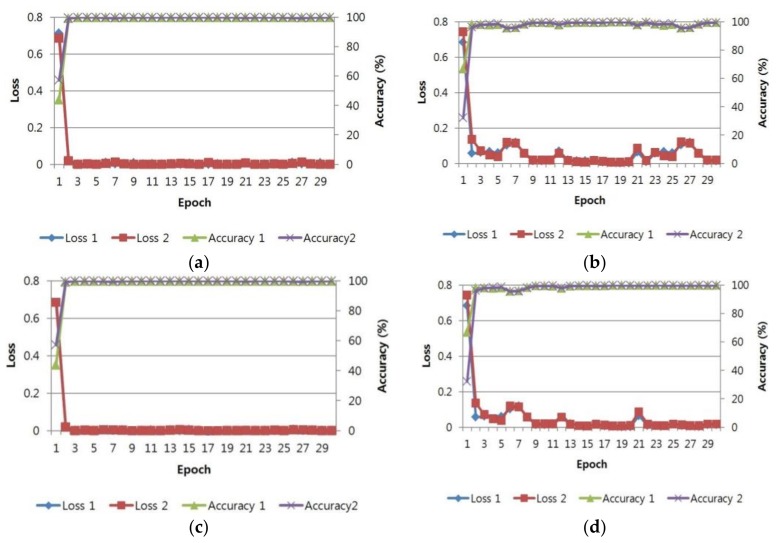
Accuracy and loss during CNN training in two-fold cross validation: (**a**,**c**) accuracy and loss in two-fold cross validation for NIR image training and validation datasets, respectively; and (**b**,**d**) accuracy and loss in two-fold cross validation for the thermal image training and validation datasets, respectively. In (**a**–**d**), “loss 1” and “accuracy 1” are from the first-fold validation, respectively. “Loss 2” and “accuracy 2” are from the second-fold validation, respectively.

Figure 9 illustrates the loss and accuracy per epoch number in the training and validation procedures. As the number of epoch increased, the loss and accuracy converged to almost 0% and 100%, respectively. This indicates successful CNN training without overfitting.

### 5.4. Testing of the Proposed CNN-Based Classification of Smooth and Aggressive Driving Emotion

Testing performance was evaluated by defining aggressive and smooth driving emotions as positive and negative data, respectively. Based on this definition, true positives (TP) (cases where the positive data is correctly identified) and true negatives (TN) (cases where the negative data is correctly identified) were defined. In addition, false negatives (FN) (cases where the positive data is incorrectly identified as negative) and false positives (FP) (case where the negative data is incorrectly identified as positive) were defined. Then, the true negative rate (TNR) (100–false positive rate (FPR) (%)) and true positive rate (TPR) (100–false negative rate (FNR) (%)) were calculated.

The first experiment compared the accuracy between the VGG face-16 model proposed in this research and AlexNet [35], which had fewer layers. Table 6 presents the structures of VGG face-16 (i.e., fine tuning) and AlexNet. AlexNet uses a larger filter and consists of five convolutional layers and three fully connected layers. Because this research aimed to distinguish two classes driving emotions (smooth and aggressive), the final, third, FCL consisted of two nodes.

Table 7 and Table 8 present TPR, TNR, FNR, and FPR in the confusion matrices of the VGG face-16 model and AlexNet. The actual and predicted items indicate the ground-truth emotion and the estimated emotion, respectively, by our method. As shown in Table 7 and Table 8, the proposed VGG face-16 model (i.e., fine tuning) has higher accuracy than AlexNet.

As mentioned in Section 4.3, CNN scores of NIR and thermal images, based on the proposed VGG face-16 model, were fused at the score-level to improve the classification accuracy of smooth and aggressive driving emotions. The experimental results show that the fusion by the weighted SUM rule produced the highest accuracy, and the optimal weights for NIR and thermal CNN scores were 2 and 1, respectively. As shown in Table 7 and Table 9, score-level fusion enhanced the classification accuracy.

**Figure 10:**
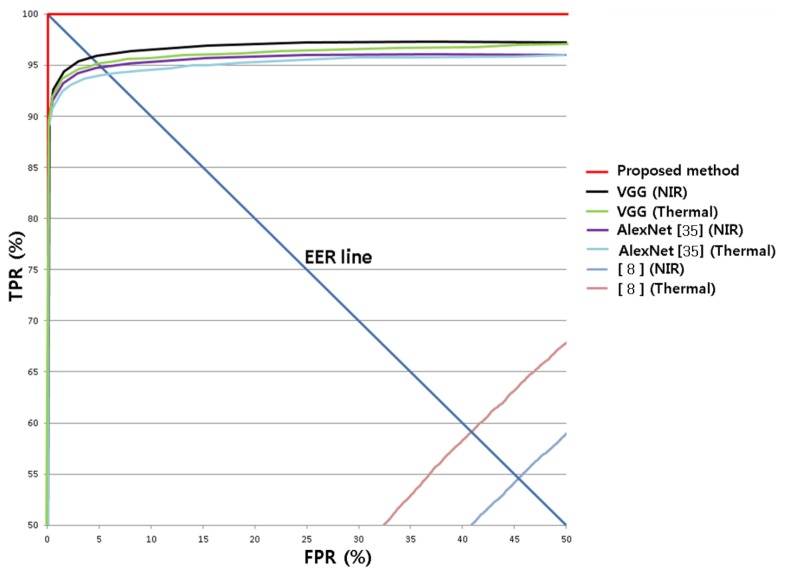
Comparisons of ROC curves of proposed and previous methods. “VGG” represents the VGG-face 16 (i.e., fine tuning).

Figure 10 compares performances by using the receiver operation characteristic (ROC) curves. The horizontal and vertical axes indicate FRP and TPR, respectively. Figure 10 also compares the classification accuracy obtained by using the HOG with a modified Hausdoff distance [8]. The graphs show the average of two results obtained from second fold cross-validation. The CNN-based score-level fusion proposed in this research had the highest accuracy.

Based on TP, TN, FP, and FN, the following four criteria were used to measure accuracy [53].

(7) $$\mathrm{Positive}\;\mathrm{predictive}\;\mathrm{value}\;\left(\mathrm{PPV}\right)=\frac{\#\mathrm{TP}}{\#\mathrm{TP}+\#\mathrm{FP}},$$

(8) $$\mathrm{TPR}=\frac{\#\mathrm{TP}}{\#\mathrm{TP}+\#\mathrm{FN}},$$

(9) $$\mathrm{Accuracy}\;\left(\mathrm{ACC}\right)=\frac{\#\mathrm{TP}+\#\mathrm{TN}}{\#\mathrm{TP}+\#\mathrm{TN}+\#\mathrm{FP}+\#\mathrm{FN}},$$

(10) $$F\_\mathrm{score}=2\cdot \frac{\mathrm{PPV}\cdot \mathrm{TPR}}{\mathrm{PPV}+\mathrm{TPR}}\;,$$

where, # means the number of cases. The maximum and minimum values of PPV, TPR, ACC, and F_score were 0% and 100%, respectively. These values indicate the lowest and highest accuracy, respectively. PPV is same as precision and TPR is identical to recall [53].

In addition, we measured the accuracy using the whole cropped face as input to the CNN. In Figure 1, the whole cropped faces from NIR and thermal images were respectively used as inputs to left and right CNN models. For that, we performed training and testing of CNN models again. Because VGG face-16 shows higher accuracy than AlexNet, VGG face-16 was used for these experiments. The experimental results showed that the PPV, TPR, ACC, and F_score of the method using the whole cropped face (weighted SUM rule) were 87.31%, 87.28%, 87.29% and 87.29%, respectively. The PPV, TPR, ACC, and F_score of the method using the whole cropped face (weighted PRODUCT rule) were 85.48%, 85.45%, 85.48%, and 85.46%, respectively, as shown in Table 10. From this, we can confirm that our method, based on the selected ROI, showed the better accuracy than that based on the whole cropped face.

As another comparison, we compared the accuracies from our method with those by multi-channel-based methods [54,55]. For this, we performed training and testing of the CNN model, again. Because VGG face-16 shows higher accuracy than AlexNet, VGG face-16 was used for these experiments. The experimental results showed that the PPV, TPR, ACC, and F_score of the method using these multi-channel images were 83.24%, 83.27%, 83.26%, and 83.25%, respectively, as shown in Table 10. From this, we can confirm that our method showed better accuracy than multi-channel-based methods [54,55]. As a whole, the proposed method from this research produced the highest accuracy, as shown in Table 10.

Most previous research on facial expression recognition using the information of RGB pixels values was performed to recognize the disguised (pretended) expressions by actors. However, we aimed to measure real emotion (not disguised or not pretended expression) of drivers. Therefore, we used information from both NIR and thermal images. 

As the next experiment, we measured the dynamic changes in the NIR and thermal face images which were captured during aggressive or smooth driving (not using the images captured while watching neutral images). Our experiments showed that dynamic changes were similar during both aggressive and smooth driving. Consequently, the average classification accuracy of aggressive and smooth driving based on the dynamic change was about 62.3%, which is much lower than that shown by our method in Table 7, Table 8 and Table 9. The reason why the dynamic change showed low accuracy is that there was no significant difference in the dynamic changes in thermal images.

Temporal modeling as a 3D cube or a recurrent neural network (or LSTM) can be considered for emotion recognition. However, this is based on the dynamic changes in successive images, which increases the complexity of system structure and training and processing time.

As the next experiment, we performed the experiments based on 10-fold cross validation. As shown in Table 11, the results from our method and previous works based on 10-fold cross validation were similar to those based on two-fold cross validation of Table 10, and we can confirm that our method outperforms other methods and previous works.

As the last experiment, we measured the human accuracies of five participants who did not take part in our experiments. Each participant manually discriminated between aggressive and smooth driving emotion after looking at the videos of NIR and thermal face images which were used for testing in our experiments. Sufficient explanations for our experiments were provided to all the participants, and we obtained written consent from all the participants before experiments.

Experimental results showed that the PPV, TPR, ACC, and F_score from human observation (human accuracies) were 76.29%, 76.32%, 76.33%, and 76.31%, respectively. By comparing the accuracies of Table 10 and Table 11, we can confirm that our method based on CNN shows the better accuracy than that by human observation. This is, because humans failed to observe tiny and fine changes in the facial images, whereas CNN could successfully extract these changes as features for emotion recognition.

## 6. Conclusions

In this research, we proposed a CNN-based method for detecting a driver’s aggressive driving emotion. The proposed CNN model uses a driver’s facial images obtained with an NIR light camera and a thermal camera. CNN scores of the images simultaneously taken by with the NIR and thermal cameras were fused at score-level to improve accuracy. The experiment with our own database showed that the proposed method produced high classification accuracy for drivers’ emotions for aggressive and smooth driving, indicating better performance compared to conventional methods. Although the accuracy with our method is close to 100%, those by other methods and previous works [8,54,55] are in the range of about 54% to about 95%, as shown in Table 10 and Table 11. From this, we can confirm the superiority of our method and that our database is not too simple. Because there is no previous open database for drivers’ emotion recognition, we would like to collect a new database while driving in an actual vehicle in real traffic. Then, we would have to test this additional database in order to check the robustness of our method in various databases. Moreover, apart from aggressive driving detection, we also expect that the proposed CNN-based method would be useful not only for detecting driver fatigue and drunken driving, but also for recognizing a broader range of emotional states.

## Figures and Tables

**Table 1:** Comparison of proposed and previous works.

Category		Methods		Advantage	Disadvantage
Gyro-sensor and accelerometer-based method		Aggressive driving detection [2,3,4,5,6]		An accelerometer and a gyro-sensor in a smart phone are used. Accordingly, no device needs to be purchased or installed. Highly portable. Because the motion of a vehicle is directly observed, it can be closely correlated to aggressive driving.	Depending on the performance of a GPS receiver, data values can be inaccurate. This method is not applicable in GPS-unavailable areas. The error rate of aggressive driving detection increases on winding and mountain roads.
Voice-based method		Detection of a driver’s emotion based on voice or car–voice interaction [12,13]		Data acquisition using inexpensive sensor.	Surrounding noise influences performance.
Bio-signal-based method		Various bio-signals, including ECG, EEG, and pulse, are measured to recognize a driver’s emotion or fatigue [14,15,16]		Bio-signals that cannot be detected by the naked eye are detected at high speed. Because bio-signals are used as input data, physiological changes can be detected.	Sensors can be detached by a driver’s active motion. The attachment of sensors may cause discomfort. Expensive sensors are used.
Camera-based method	Using single camera	Using visible light camera	Yawning detection [10]	An inexpensive camera is used.	Physical characteristics that cannot be observed by the naked eye are not detected. Measurement becomes difficult at night or in tunnels.
		Using thermal camera	Driver’s emotion recognition [8]	Night photography is possible without a separate light source. Subtle physical signals related to specific emotions, which cannot be caught by visible light or NIR cameras, can be detected.	The camera is expensive compared to visible light or NIR cameras. The camera is less effective for detecting facial regions and facial feature points than with NIR cameras.
	Using multiple cameras	Using dual NIR cameras	Percentage of eye closure over time (PERCLOS)- and average eye-closure speed (AECS)-based detection of driver fatigue [9]	In cases where more than two cameras are used, driver fatigue is detected over a wide range. NIR light makes detection possible at night or in a tunnel.	Physical characteristics that cannot be observed by the naked eye are not detected.
		Using NIR and thermal cameras	Aggressive driving emotion detection-based convolutional neural networks (CNN) (Proposed method)	Thermal cameras can measure temperature changes in a driver’s body, which cannot be checked by the naked eye, whereas, a NIR camera can detect facial feature points and measure their changes. An intensively trained CNN is robust in various environmental and driver conditions.	The use of two cameras increases algorithm complexity and processing time. Intensive CNN training is required.

**Table 2:** CNN architecture used in our research (i.e., Conv, ReLU, and Pool mean convolutional layer, rectified linear unit, and max pooling layer, respectively).

Layer Type		Number of Filters	Size of Feature Map	Size of Kernel	Number of Stride	Number of Padding
Image input layer			224 (height) × 224 (width) × 3 (channel)			
Group 1	Conv1_1 (1st convolutional layer)	64	224 × 224 × 64	3 × 3	1 × 1	1 × 1
	ReLU1_1		224 × 224 × 64			
	Conv1_2 (2nd convolutional layer)	64	224 × 224 × 64	3 × 3	1 × 1	1 × 1
	ReLU1_2		224 × 224 × 64			
	Pool1	1	112 × 112 × 64	2 × 2	2 × 2	0 × 0
Group 2	Conv2_1 (3rd convolutional layer)	128	112 × 112 × 128	3 × 3	1 × 1	1 × 1
	ReLU2_1		112 × 112 × 128			
	Conv2_2 (4th convolutional layer)	128	112 × 112 × 128	3 × 3	1 × 1	1 × 1
	ReLU2_2		112 × 112 × 128			
	Pool2	1	56 × 56 × 128	2 × 2	2 × 2	0 × 0
Group 3	Conv3_1 (5th convolutional layer)	256	56 × 56 × 256	3 × 3	1 × 1	1 × 1
	ReLU3_1		56 × 56 × 256			
	Conv3_2 (6th convolutional layer)	256	56 × 56 × 256	3 × 3	1 × 1	1 × 1
	ReLU3_2		56 × 56 × 256			
	Conv3_3 (7th convolutional layer)	256	56 × 56 × 256	3 × 3	1 × 1	1 × 1
	ReLU3_3		56 × 56 × 256			
	Pool3	1	28 × 28 × 256	2 × 2	2 × 2	0 × 0
Group 4	Conv4_1 (8th convolutional layer)	512	28 × 28 × 512	3 × 3	1 × 1	1 × 1
	ReLU4_1		28 × 28 × 512			
	Conv4_2 (9th convolutional layer)	512	28 × 28 × 512	3 × 3	1 × 1	1 × 1
	ReLU4_2		28 × 28 × 512			
	Conv4_3 (10th convolutional layer)	512	28 × 28 × 512	3 × 3	1 × 1	1 × 1
	ReLU4_3		28 × 28 × 512			
	Pool4	1	14 × 14 × 512	2 × 2	2 × 2	0 × 0
Group 5	Conv5_1 (11th convolutional layer)	512	14 × 14 × 512	3 × 3	1 × 1	1 × 1
	ReLU5_1		14 × 14 × 512			
	Conv5_2 (12th convolutional layer)	512	14 × 14 × 512	3 × 3	1 × 1	1 × 1
	ReLU5_2		14 × 14 × 512			
	Conv5_3 (13th convolutional layer)	512	14 × 14 × 512	3 × 3	1 × 1	1 × 1
	ReLU5_3		14 × 14 × 512			
	Pool5	1	7 × 7 × 512	2 × 2	2 × 2	0 × 0
	Fc6 (1st FCL)		4096 × 1			
	ReLU6		4096 × 1			
	Dropout6		4096 × 1			
	Fc7 (2nd FCL)		4096 × 1			
	ReLU7		4096 × 1			
	Dropout7		4096 × 1			
	Fc8 (3rd FCL)		2 × 1			
	Softmax layer		2 × 1			
	Output layer		2 × 1			

**Table 3:** Image database.

	NIR Images		Thermal Images	
	Smooth Driving	Aggressive Driving	Smooth Driving	Aggressive Driving
Number of images	29,463	29,463	29,463	29,463

**Table 4:** *p*-Value, Cohen’s *d* value, and effect size of five features between smooth and aggressive driving.

	**DLRL**		**DULL**		**HR**	
	**Smooth**	**Aggressive**	**Smooth**	**Aggressive**	**Smooth**	**Aggressive**
*p*-value	0.2582		0.1441		0.7308	
Cohen’s *d* value	0.4233		0.5487		0.1325	
Effect size	medium		medium		Small	
	**EBR**		**EM**			
	**Smooth**	**Aggressive**	**Smooth**		**Aggressive**	
*p*-value	0.9490		0.6715			
Cohen’s *d* value	0.0236		0.1565			
Effect size	Small		Small			

**Table 5:** *p*-Value, Cohen’s *d* value and effect size for pixel values of NIR and thermal ROIs between smooth and aggressive driving.

	**Left Eye**		**Right Eye**		**Mouth**	
	**Smooth**	**Aggressive**	**Smooth**	**Aggressive**	**Smooth**	**Aggressive**
*p*-value	0.0046		0.0123		0.0021	
Cohen’s *d* value	1.1234		0.9842		1.2355	
Effect size	Large		Large		Large	
	**Middle of Forehead**		**Left Cheek**		**Right Cheek**	
	**Smooth**	**Aggressive**	**Smooth**	**Aggressive**	**Smooth**	**Aggressive**
*p*-value	0.0139		0.0450		0.0476	
Cohen’s *d* value	0.9770		0.7662		0.7565	
Effect size	Large		Large		Large	

**Table 6:** CNN models for comparison. ConvN is the filter of N × N size (e.g., Conv3 represents a 3 × 3 filter).

Net Configuration	VGG face-16 (Fine Tuning) (Proposed Method)	AlexNet
# of layers	16	8
Filter size (# of filters)	Conv3 (64) Conv3 (64)	Conv11 (96)
Pooling type	MAX	MAX
Filter size (# of filters)	Conv3 (128) Conv3 (128)	Conv5 (256)
Pooling type	MAX	MAX
Filter size (# of filters)	Conv3 (256) Conv3 (256) Conv3 (256)	Conv3 (384)
Pooling type	MAX	-
Filter size (# of filters)	Conv3 (512) Conv3 (512) Conv3 (512)	Conv3 (384)
Pooling type	MAX	-
Filter size (# of filters)	Conv3 (512) Conv3 (512) Conv3 (512)	Conv3 (256)
Pooling Type	MAX	MAX
Fc6 (1st FCL) Fc7 (2nd FCL) Fc8 (3rd FCL)	409 6409 62	409 6409 62

**Table 7:** Classification accuracies by proposed VGG face-16 model (%).

**VGG Face-16 Model (NIR Images)**						
**Actual**	**Predicted**					
	**First fold**		**Second fold**		**Average**	
	**Aggressive**	**Smooth**	**Aggressive**	**Smooth**	**Aggressive**	**Smooth**
Aggressive	95.913	4.087	95.941	4.059	95.927	4.073
Smooth	4.06	95.94	4.057	95.943	4.0585	95.9415
**VGG Face-16 Model (Thermal Images)**						
**Actual**	**Predicted**					
	**First fold**		**Second fold**		**Average**	
	**Aggressive**	**Smooth**	**Aggressive**	**Smooth**	**Aggressive**	**Smooth**
Aggressive	95.859	4.141	94.773	5.227	95.316	4.684
Smooth	5.143	94.857	5.217	94.783	5.18	94.82

**Table 8:** Classification accuracies by AlexNet model (unit: %).

**AlexNet (NIR Images)**						
**Actual**	**Predicted**					
	**First fold**		**Second fold**		**Average**	
	**Aggressive**	**Smooth**	**Aggressive**	**Smooth**	**Aggressive**	**Smooth**
Aggressive	94.885	5.115	94.931	5.069	94.908	5.092
Smooth	5.057	94.943	5.080	94.920	5.0685	94.9315
**AlexNet (Thermal Images)**						
**Actual**	**Predicted**					
	**First fold**		**Second fold**		**Average**	
	**Aggressive**	**Smooth**	**Aggressive**	**Smooth**	**Aggressive**	**Smooth**
Aggressive	94.076	5.924	94.008	5.992	94.042	5.958
Smooth	5.964	94.036	5.884	94.116	5.924	94.076

**Table 9:** Classification accuracies by score-level fusion, based on weighted SUM rule of the proposed method (%).

Actual	Predicted					
	First fold		Second fold		Average	
	Aggressive	Smooth	Aggressive	Smooth	Aggressive	Smooth
Aggressive	99.955	0.045	99.972	0.028	99.9635	0.0365
Smooth	0.053	99.947	0.027	99.973	0.04	99.96

**Table 10:** Comparisons of positive predictive value (PPV), true positive rate (TPR), accuracy (ACC), and F_score of the proposed and previous methods. “VGG” represents VGG-face 16 (i.e., fine tuning) (%).

	PPV	TPR	ACC	F_Score
Proposed	99.96	99.97	99.96	99.97
VGG (NIR)	95.94	95.92	95.93	95.93
VGG (Thermal)	95.08	95.07	95.07	95.08
AlexNet (NIR)	94.92	94.91	94.92	94.91
AlexNet (Thermal)	94.07	94.06	94.06	94.07
Method using whole face (weight SUM rule)	87.31	87.28	87.29	87.29
Method using whole face (weight PRODUCT rule)	85.48	85.45	85.48	85.46
Multi-channel-based method [54,55]	83.24	83.27	83.26	83.25
[8] (NIR)	54.01	54.1	54.05	54.05
[8] (Thermal)	58.39	58.29	58.34	58.33

**Table 11:** Comparisons of PPV, TPR, ACC, and F_score of proposed and previous methods based on 10-fold cross validation. “VGG” represents VGG-face 16 (i.e., fine tuning) (%).

	PPV	TPR	ACC	F_Score
Proposed	99.94	99.95	99.95	99.94
VGG (NIR)	95.87	95.85	95.85	95.86
VGG (Thermal)	95.11	95.1	95.1	95.1
AlexNet (NIR)	94.85	94.87	94.86	94.86
AlexNet (Thermal)	94.12	94.1	94.11	94.11
Method using whole face (weight SUM rule)	86.11	86.09	87.1	86.1
Method using whole face (weight PRODUCT rule)	85.28	85.25	87.27	85.26
Multi-channel-based method [54,55]	82.19	82.17	82.17	82.18
[8] (NIR)	55.21	55.24	54.22	55.22
[8] (Thermal)	59.28	59.25	59.27	59.26
